# A comparative analysis of BCL-2 family

**DOI:** 10.6026/97320630015299

**Published:** 2019-04-15

**Authors:** Shouhartha Choudhury

**Affiliations:** 1 Department of Biotechnology, Assam University, Silchar-788011, Assam, India

**Keywords:** BCL-2 family, pro-apoptotic, anti-apoptotic, divergent genes

## Abstract

The BCL-2 family is conserved in evolution and shares a BCL-2 homology domain. It promotes and inhibits apoptosis. It is also known that apoptosis has a major role in effective cancer treatment. Therefore, it is of interest to document information related to the BCL-2 family of proteins for analysis by prediction tools. Hence, insights from a prediction based comparative functional analysis of 108 genes in this family are documented.

## Background

One of the biggest challenges for evolutionary biologists in the post-genomic era has been a question of how phenotypic diversity arises. Novel genes can rapidly integrate into existing and effectively drive the evolution of phenotypes 1. It is not only the genes themselves but also when, how and in what combination they are expressed in the cells is critical. The family-wise classification is an essential component for the identification of a specific gene involved in the genome. The genome sequence of ancestral species cannot be gleaned from contemporary databases. The coding and non-coding regions are amplified in a different manner, thus creating considerable variability between taxa and species. The coding region, duplication or retro transposition processes give rise to isoforms often possessing new innovative function. In evolution, genes having a common origin are defined 'homologous', which subdivided into 'orthologous' if divergence due to speciation and 'paralogous' if they are generated by duplication. Paralogous genes are clustered in gene families, which often differ in the number, and role of their components in different species, even closely related ones. What matters are to know which functions are conserved in the various organisms and which innovations are created in response to precise metabolic needs in a given species? 2. The lack of ancestral sequences tracing the evolutionary history of the genes and their cellular process is a bottleneck. The chronological order of discovery profoundly influences nomenclature and the interpretation of evidence, and also their notorious hurdles in obtaining reliable alignments of divergent or dissimilar sequences. Despite limitations, comparisons across species have catapulted forward our understanding of biological processes exemplified by BCL-2 family 3, 4. Thus, crossspecies comparisons needs to be captured and understood to broaden our biological knowledge of BCL-2 family and their central roles in cancer formation. Encoded BCL-2 founding member of the eponymous protein family was discovered more than 20 years ago at the chromosomal breakpoint t (14; 18) translocation in human follicular B-cell lymphomas 4-8. BCL-2 family proteins are evolutionary conserved and share BCL-2 homology (BH) domains. The encoded BCL-2 gene localizes to intracellular membranes such as endoplasmic reticulum and mitochondria, and other family members translocated cytoplasm to mitochondria following a cell death stimulus. The prototypical BCL-2 gene was originally identified at chromosome translocation breakpoint in human and was subsequently shown to promote tumorigenesis by inhibiting cell death rather than by promoting cell-cycle progression. BCL-2 family of proteins are classified anti-apoptotic, pro-apoptotic and divergent (data available with authors). The traditional view, antideath BCL-2 family members in healthy cells hold pro-death BCL-2 family members. BH3-only domain inactivates the protective BCL-2 proteins and forcing them to release their pro-death partners. The pro-death BCL-2 family protein homos-oligomerize to create pores in the mitochondrial outer membrane, resulting in cytochrome c release into the cytoplasm, which leads to activation and cell death. An alternative model suggests anti-death BCL-2 proteins bind and inhibit a subset of BH3 domains directly induce oligomerization of BAX or BAK. The computational methods and subjective interpretations of the sequence similarities have expanded BCL-2 family beyond justifiable limits. Biases in the BCL-2 family nomenclature extend beyond amino acid sequence analysis. Assignment to anti-death, pro-death and divergent groups is challenging for cell death-related phenotypes and exhibit anti-death or pro-death activity in different conditions or cell types. The cell death program is rather simple and few gene products essentially carry it out. In higher eukaryotes, complex nature and their different endogenous environmental death stimuli, components have evolved into protein families whose encoded gene act in different cell types and different intracellular locations. Classification with information on given protein family and their encoded gene such as primary sequence, conserved domain, motifs, chromosome location, evolution and gene expression will contribute a better understanding of the function of each gene in the genome. This turn can guide experimental and practical applications. In this study, we report the classification of BCL-2 family involved in the cell death program in eukaryotes. The apoptotic process or cell death program is crucial for organism survival and is conserved in evolution. It�s an essential component of animal development important for establishment and maintenance of tissue architecture processes based upon the formation and removal of specific structures 9-12. This flexibility of the primordial structures can adapt to different functions at various stages in life or in different sexes. In this report, we performed bioinformatics and computational analysis of major BCL-2 family components identified so far. 

## Methodology

### Primary sequence and database

Primary query sequence (BCL-2) information retrieved from the different databases such as NCBI, UniProt, EMBL, GenBank and performed web base application SMART for identification of specific domain. Pfam was searched for retrieving protein family information. PROSITE performed for the identification domain, family and functional sites as well as associated pattern and profile. PROCHECK examine the stereo chemical quality of the primary peptide sequence. The genome sequences were downloaded from genomic data in different specialized databases (NCBI, Ensemble, and TIGR).

### Standalone tools and GO annotation

HMMER executed using multiple sequence alignments of the specific BCL domain as a profile search in an individual genome. A statistical algorithm searching sequence homologs, making multiple sequence alignment of a specific domain as a profile search, its implements methods using probabilistic models called profile is hidden Markov model. BLAST standalone executed for the identification of homologs gene. The BLAST2GO performed for the accuracy of novel sequences in the genome, a bioinformatics and computational tool for high-throughput gene annotation of novel sequence data. The functional information retrieves via Gene Ontology (GO) annotation, a controlled vocabulary of the functional attributes.

### Domain, motif, and phylogeny

MSA (Multiple Sequence Alignment) of the multiple hits of query gene (BCL-2) analysis was carried out with a web-based tool MultAlin for identification of the conserved BCL domain in Homo sapiens, Pan Troglodytes and Mus musculus. MSA is a multiple sequence alignment methods to calculate the best match of homologs sequences. The identification of the molecular evolutionary relationship between anti-apoptotic, pro-apoptotic and divergent groups, we performed MEGA7 for the phylogenetic tree using Neighbor-Joining Methods. The MEME suite is a computational tool for discovery and analysis of sequence motifs; we performed a MEME web-based tool for retrieving motifs composition in the novel sequences.

### Gene expression and chromosome location

Expression analysis of the query gene, we initialized GENEVESTIGATOR is a high-performance search engine for gene expression of different biological contexts. Gene card: Is a database of human genes that provides genomic, proteomic, transcriptomic, genetic and functional information on all known and predicted human genes, we retrieved chromosome location using gene card.

## Results 

**Figure 1:**
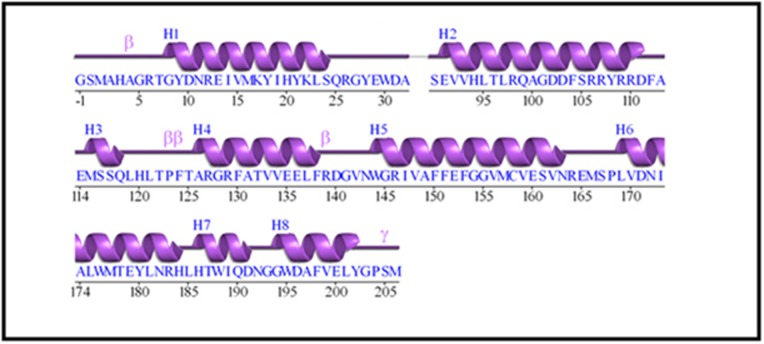
Primary sequence information for BCL-2 (GenBank Id: NM_000633.2)

**Figure 2:**
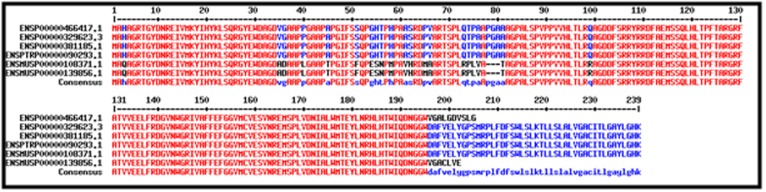
Multiple sequence alignment (conserved BCL domain with high consensus)

**Figure 3:**
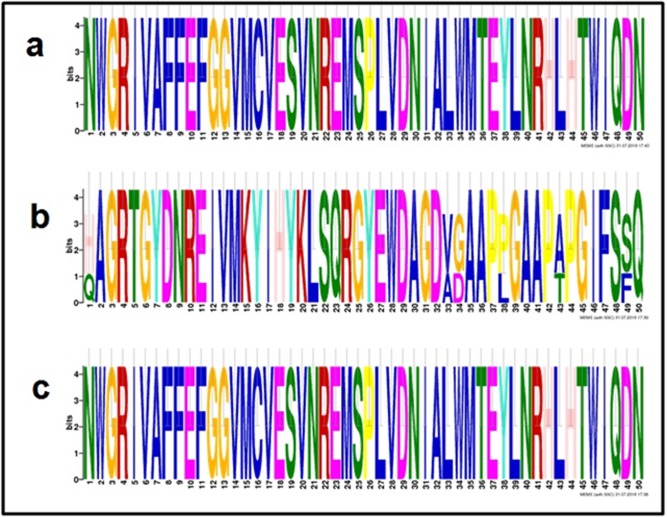
Motifs in BCL-2 family of proteins

**Figure 4:**
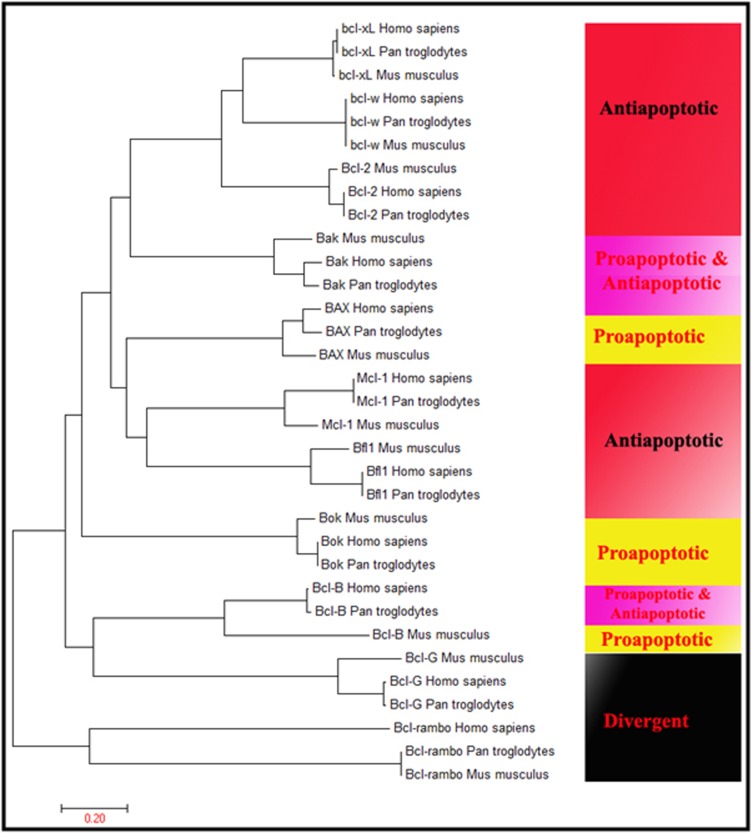
Phylogenetic tree: (a) Anti-apoptotic, (b) Pro-apoptotic and (c) Divergent

**Figure 5:**
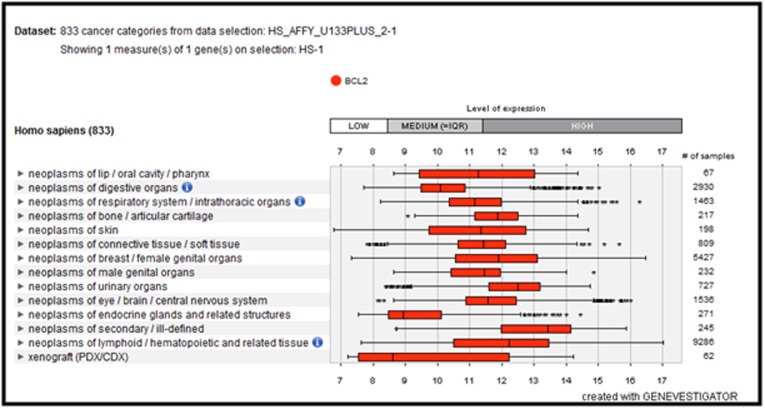
Expression analysis of BCL-2 gene: Highly expressed in neoplasms of secondary ill-defined, skin, breast, female genital organs, lymphoid, hematopoietic tissue, eye, brain, and central nervous system

**Figure 6:**
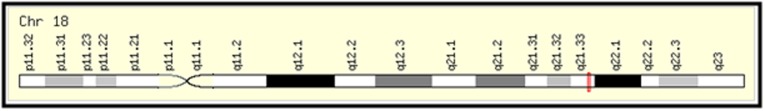
BCL-2 located in chromosome 18

BCL-2 family in Homo sapiens, Pan Troglodytes and Mus musculus were identified in this study. The query gene BCL-2 a founding member of the BCL-2 family inside the protein with seven alpha helices, having two hydrophobic helices, is flanked by five amphipathic helices (Figure 1). However, some in cases domain is questionable and size and borders cannot be precisely defined. Mutagenesis experiments prove that the BH1, BH2, and BH3 domains deeply influence homo and hetero-dimerization. BH4 domain stabilizes the structure of the hydrophobic groove. BCL-2 family clearly shows that proteins having a similar structure can be adapted to different roles, sometimes opposite one with few changes in their primary or secondary structure 13. The primary nucleotide and peptide sequence demonstrated the length of the sequence and composition of 720 nucleic acids translated to 239 amino acids within 99 amino acids binding to the DNA. HMMER results show a total number of BCL domain consisting of 53, 25 and 34 in Homo sapiens, Pan Troglodytes and Mus musculus, respectively. BH4 domain (central domain) as profile search and obtain more BH4 and BCL domain involved gene encoded in BCL-2 family. In addition, we observed some other domains are involved, which is not characteristic of BCL-2 family; therefore we did not consider them in the current study. Standalone BLAST results demonstrated the number of homologs sequences in the genome of all organisms studied in this analysis. Gene ontology annotation summary demonstrated the accuracy of BCL and BH4 domain involved. Form our analysis it was observed that the total number of BCL-2 family encoded gene 51, 24 and 33 in Homo sapiens, Pan Troglodytes and Mus musculus respectively. The comparative and functional analysis obtained specific BH4 and BCL domain gene encoded in the BCL-2 family (data available with authors) and undertook a survey of recognizable BCL-2 family. The number of encoded genes i.e. 51, 24 and 33 in Homo sapiens, Pan Troglodytes and Mus musculus (data available with authors) respectively and investigated 108 genes in all organisms (data available with authors). We further classified them into three groups namely anti-apoptotic, proapoptotic and divergent (data available with authors). Multiple sequence alignment (MSA) of the sequences examined has conserved domains (Figure 2) with their specific motifs (Figure 3). The phylogenetic tree branching diagram defines the evolutionary relationship between Homo sapiens, Pan Troglodytes and Mus musculus. Particular clades represent anti-apoptotic, pro-apoptotic and divergent genes in all organisms (Figure 4). Gene expression analysis has shown that the BCL-2 gene is highly expressed in neoplasms of lip/oral cavity/pharynx, respiratory system/intrathoracic organs, bone/articular cartilage, skin, connective tissue, breast/female genital organs, urinary organs, lymphoid/hematopoietic tissue, eye/brain/central nervous system (Figure 5). Chromosome localization study demonstrated BCL-2 located in chromosome 18 (q21.33) (Figure 6). In order to determine the family expansion in different molecular evolutionary lineages defined in phylogeny. In this study, we conducted a comprehensive survey of the BCL-2 family for understanding the molecular evolutionarily conserved mechanisms.

##  Discussion

Specific genes encoded in the BCL-2 family in all organisms genome are collected (data available with authors). Chemoresistance is a major obstacle for successful treatment of cancer; therefore the identification of regions in the genome associated with acquired resistance to therapeutic remedy is essential. Comparative genomics studies revealed the region of gain or loss of DNA that were characteristic of drug-resistant cell line: i.e. differences their drug-sensitive parental cell line. Clinically, primary human melanoma revealed nearest neighbour linkage MITF (micro-phthalmia-associated transcription factor) and BCL-2 was rearranged in major breakpoint cluster region and joined into immunoglobin heavy chain in follicular lymphoma. The oncogene BCL-2 in leukemia cells in a patient with antagonistic Prolymphocytic leukemia has an abnormal karyotype; it remains to establish somatic mutations alter in lymphoma. The lymphoma cell line with complex translocation rearrangement pre-treated BCL-2 expression was specifically associated with distant metastasis of the patients whose primary tumors positive 4. BCL-2 family share homology clustered within four conserved regions BH1, BH2, BH3 and BH-4 control the ability of proteins to dimerize function as regulators of apoptosis. BCL-XL confer a level of drug resistance revealed overexpression of gene contributes to the cisplatinresistant phenotypes in Osteosarcoma cell system. BCL-XL correlated number of apoptotic lymphoma cell by terminal deoxytransferase- catalyzed nick-end labelling. BCL-XL expression as a prognostic marker in follicular lymphoma should be considered; an expression of BCL-2 was significantly enhanced in cutaneous lesions of adult and pediatric patients. BCL-XL slightly increased in pediatric, but not in adult patients with mastocytosis. BCL-XL inhabits withdrawal cell death upon growth factor. Downregulation of intimal cell BCL-XL induced apoptosis and regression of vascular lesions. These results suggested apoptosis regulatory BCL-XL are critical determinants of intimal lesion formation and targeted apoptosis may be a novel therapy for intimal vascular disease. Down-regulation of BCL2L2 (BCL-W) sensitized VP-16 resistant ovarian cancer cell line and NF-kappaBmediated upregulation of BCL-XL and BCL-W expression in glioma cells. Increase cellular resistance to cytotoxic therapy-induced apoptosis. BCL-XL and BCL-W foster malignant glioblastoma cell survival. The BCL-W in testis appears to restrict elongating spermatids and Sertoli cells depletion of BCL-XL or BCL-W antagonized TWEAK protective on glioma cells. Apoptosis regulators BCL-W decrease irradiated T-cells. The therapeutic effects of gene transfer mediated elevation suggesting perturbation of BAK-mediated apoptosis contribute the pathogenesis of gastric cancer. Furthermore, BCL-XL and BID aligning BAK-mediated BH3 motifs are known as BCL-XL and BAK (BH3 complex). The activation of multi-domain pro-apoptotic BAK appears to mitochondrial dysfunction cell death in response to diverse stimuli. The BH3 domain control specificity and regulate MCL-1 and BAKmediated apoptosis. MCL-1 maintains BAK inactive state and loss MCL-1 activation, perhaps replication stress induces in infected cells may be required to initiate the apoptotic response. BAK/BAXmediated mitochondrial outer-membrane drives cell death during development and tissue homeostasis in the human. The patients with normal karyotype showed a higher frequency of BCL2A1 in abnormal karyotype and cancer cell lines demonstrated hematopoietic malignancies and melanoma. The identification of novel minor histo-compatibility antigens (mHAgs) encoded by two separate single nucleotide polymorphisms (SNP) in a single gene, BCL2A1 restricted by human histo-compatibility leukocyte antigen HLA (human leukocyte antigen) the most common HLA-A allele in Japanese. BCL2A1 reported in hematopoietic cells and possess nonsynonymous nucleotide. The newly identified HLA-A24-restricted minor histocompatibility antigen epitope derived BCL2A1 and ACC-1 in patients receiving HLA genotypically matched unrelated bone marrow transplant. The myocytes reveal that the BCL-2 protects the cell against apoptosis in heart patients with cardiac failure, whereas labelling with BAX that promote apoptosis remains constant. BAX is an apoptosis-encoded protein (BCL-2 associate X protein) participates in cell death during normal development. The role of BAX in drug-induced apoptosis in human colorectal cancer cells identified lack function of BAX; in contrast absence of BAX completely abolished apoptotic response. BAX also render colorectal cancer resistant TRAIL/Apo2L-mediated radiosensitization. Inactivation of BAX and BAK promote epithelial solid tumor growth and resistance to chemotherapy. Clear contrast of BAX is frequently inactivated correlates to pore prognosis; there are no significant differences of BAX between goiters or adenoma. The elevated BAX in patients with thyroid carcinoma compared to the patients of adenoma in goiters. The BAX represent a prognostic indicator of the patients with ovarian cancer and combine evaluation of BAX and BCL-2 may provide additional prognostic significance. In contrast, apoptosis induced staurosporine does not require protein synthesis but it is characterized by the translocation of BAX. MCL-1 has sequence similarity with BCL-2 and involved in normal development in lymphoma. MCL-1 isolated human myeloid leukemia cell line during phorbol ester-induced differentiation along monocyte. MCL-1 indicates Burkitt lymphoma subline exhibit enhance survival exposure to serum deprivation. MCL-1 related to a vascular endothelial growth factor is associated with pore outcome in non-Hodgkin's lymphoma. The mechanism of MCL-1 induced survival and transformation in the genome, the MCL-1-overexpressing B-cell lymphoma. BH3-only domain control specificity regulates MCL-1 and BAK-mediated apoptosis, we demonstrated uninfected cells BAK is complex with anti-apoptotic induce Myeloid cell leukemia 1. BCL-G consists of 327 and 252 amino acid lengths with 6 exons residues on the chromosome, and encoded protein through an alternative mRNA splicing. Novel TEL-AML1 fusion transcript involves pro-apoptotic BCL-G in the pediatric precursor of B-cell acute lymphoblastic leukemia. BCL-G significantly down-regulated in both clinical tumors and cultured prostate cancer tissues indicated that the critical initiation or progression of prostate carcinoma. The BCL-Rambo was confined to mitochondria and over-express apoptosis specifically blocked cascade inhibitor and controlling upstream events of either �death receptor� or mitochondrial pro-apoptotic. The pro-apoptotic BCLRambo designated as BCL-Rambo beta is induced in several adult human tissues such as heart, lymph node, and cervix but absent in human brain tissue, unlike BCL-Rambo is lacking BH1, BH2, and BH3 domain. The BCL2L10 (BCL-B, Boo) is a negative regulator of cell death in the human glioma cell. In addition, BCL2L10 could promote apoptosis and growth inhibitory effect in gastric cancer cell lines. The mammalian homologs Boo and BCL-XL interact with the human counterpart of Ced-4 and Apaf-1 regulate apoptosis and located to murine chromosome 9. BCL2L10 was restricted to ovary and epididymis implicating in control of ovarian atresia and sperm maturation. BH4 domain and trans-membrane (TM) domain in BCL2L10 are necessary for suppressive action on cell death. The BCL2L10 eponymous anti-apoptotic of the BCL-2 family blocks apoptosis in the mitochondria death pathway but not in the death receptor pathway 6, 14-20.

## Conclusion

Several homologous BCL-2 domains in the BCL-2 family of proteins were identified to be associated as a causal factor in cancer. Therefore, a comparative functional analysis of this family of proteins is documented in this report.

## Conflict of Interest

Authors declare no conflict of interest.
